# Understanding feedback report uptake: process evaluation findings from a 13-month feedback intervention in long-term care settings

**DOI:** 10.1186/s13012-015-0208-2

**Published:** 2015-02-12

**Authors:** Anne E Sales, Kimberly Fraser, Melba Andrea B Baylon, Hannah M O’Rourke, Gloria Gao, Tracey Bucknall, Suzanne Maisey

**Affiliations:** Faculty of Nursing, University of Alberta, Edmonton, AB Canada; Center for Clinical Management Research, VA Ann Arbor Healthcare System, Division of Nursing Business and Health Systems, School of Nursing, University of Michigan, 400 N. Ingalls Street, Ann Arbor, MI 48109-5482 USA; Faculty of Nursing, Deakin University, Melbourne, VIC Australia; Shepherd’s Care Foundation, Edmonton, AB Canada

**Keywords:** Feedback intervention, Long-term care, Quality improvement, Process evaluation

## Abstract

**Background:**

Long-term care settings provide care to a large proportion of predominantly older, highly disabled adults across the United States and Canada. Managing and improving quality of care is challenging, in part because staffing is highly dependent on relatively non-professional health care aides and resources are limited. Feedback interventions in these settings are relatively rare, and there has been little published information about the process of feedback intervention. Our objectives were to describe the key components of uptake of the feedback reports, as well as other indicators of participant response to the intervention.

**Methods:**

We conducted this project in nine long-term care units in four facilities in Edmonton, Canada. We used mixed methods, including observations during a 13-month feedback report intervention with nine post-feedback survey cycles, to conduct a process evaluation of a feedback report intervention in these units. We included all facility-based direct care providers (staff) in the feedback report distribution and survey administration. We conducted descriptive analyses of the data from observations and surveys, presenting this in tabular and graphic form. We constructed a short scale to measure uptake of the feedback reports. Our analysis evaluated feedback report uptake by provider type over the 13 months of the intervention.

**Results:**

We received a total of 1,080 survey responses over the period of the intervention, which varied by type of provider, facility, and survey month. Total number of reports distributed ranged from 103 in cycle 12 to 229 in cycle 3, although the method of delivery varied widely across the period, from 12% to 65% delivered directly to individuals and 15% to 84% left for later distribution. The key elements of feedback uptake, including receiving, reading, understanding, discussing, and reporting a perception that the reports were useful, varied by survey cycle and provider type, as well as by facility. Uptake, as we measured it, was consistently high overall, but varied widely by provider type and time period.

**Conclusions:**

We report detailed process data describing the aspects of uptake of a feedback report during an intensive, longitudinal feedback intervention in long-term care facilities. Uptake is a complex process for which we used multiple measures. We demonstrate the feasibility of conducting a complex longitudinal feedback intervention in relatively resource-poor long-term care facilities to a wider range of provider types than have been included in prior feedback interventions.

**Electronic supplementary material:**

The online version of this article (doi:10.1186/s13012-015-0208-2) contains supplementary material, which is available to authorized users.

## Background and significance

Long-term care (LTC) settings, which include nursing homes (NHs) and other facility-based settings in which individuals receive residential care for serious long-term chronic health conditions, provide care to a relatively large proportion of predominantly older, highly disabled adults across the United States and Canada. Staffing levels within LTC settings are typically lower than that in acute care, with high resident:staff ratios. The majority of direct-care staff are health care aides (HCAs, also sometimes referred to as personal care attendants) whose educational background are widely variable and who may or may not have prior experience in health care [1].

In these settings, managing and improving quality of care is challenging. Relatively few efforts at quality improvement have been documented in these settings compared to those in acute and primary care [2]. However, the increasingly widespread use of standardized resident assessment is an opportunity for feedback interventions to improve the quality of care, using readily available data that are collected for routine care. Feedback (or audit and feedback) interventions involve providing individuals or groups with information about their performance, often through describing outcomes of care. These have been used extensively in health care settings other than LTC as an approach to improve performance and quality of care [3], and overall, they have been found to be effective at changing provider behavior in desired directions, although findings continue to be mixed across settings, provider, or staff types and other factors [3].

Feedback interventions in LTC settings are relatively rare and typically involve only one type of provider, usually registered nurses [4,5]. In the few published studies to date, there has been relatively little information about how the feedback reports were distributed or how the staff who received them used the reports and whether or not they found them useful. This leaves a gap in our understanding of how these reports are used and whether or not they are actually received and processed by the people targeted to receive them.

We conducted a feedback intervention in nine long-term care units, across four facilities, in Edmonton, Alberta, Canada, between January 2009 and February 2010. The results of the summative evaluation of this intervention are reported in a companion paper [6]. Overall, the feedback intervention was not effective in changing resident outcomes across the elements reported in the feedback reports. There was a modest and statistically significant improvement in pain scores after the beginning of the intervention.

In this paper, we report on the process evaluation that we conducted concurrently with the feedback intervention to understand whether the feedback report was taken up and used by participants, one possible reason for the intervention’s lack of effectiveness. Process measures may be relevant in assessing whether or not interventions are fully implemented [7,8]. One of the most commonly observed reasons for failure of a quality improvement or knowledge translation intervention is lack of uptake of the intervention [9-13]. As a result, it is essential to understand uptake. In the most recent Cochrane review of audit with feedback interventions, uptake measurement was only reported in 36 of the 140 reports (26%) included in the review [3].

We used the Theory of Planned Behavior (TPB) [8,14] as the conceptual model for our project (Additional file 1). Our expectation was that the feedback report would affect attitudes towards the behavior but also directly affect intention to change behavior. We focused on uptake as a critical element of the intervention, and in this report of the process evaluation, we focus on describing elements related to uptake of the intervention, which we defined as receiving, reading, and understanding the reports. Discussing the reports with other staff was of particular interest because of our underlying interest in whether or not the report might change attitudes, beliefs, and particularly social norms. We were further interested in whether or not the participants found the reports useful, both generally and more specifically in changing the way they cared for residents. Finally, we were interested in their self-report of intention to change behavior for one key measure, assessing pain among residents they cared for in the next shift. Our focus on this particular aspect of care was related to the high importance placed on it by both staff in the pilot study that we conducted to develop the feedback reports, as well as by senior leaders in the two participating organizations.

### Purpose and objectives

Our objectives in conducting the process evaluation were to describe data on uptake of feedback reports, understand participants’ reactions to the reports including whether they discussed them with other staff members and found them useful, and to understand trends in participation and response over the 13 months of the intervention.

## Methods

We provide a short description of the methods for the process evaluation in this section, highlighting the methods for the process evaluation rather than the feedback intervention itself. We provide a much fuller description of methods in Additional file 1. We published the protocol for the study previously, which described our plans for conducting the study [15], and the summative results of the trial appear in a companion paper [6]. The full study was approved by the Health Research Ethics Board Committee B at the University of Alberta and by the relevant committees and decision-makers in each of the participating organizations.

The overall intervention involved 13 monthly feedback reports, described in more detail in the additional file. We conducted surveys to assess uptake of the feedback intervention 1 week after feedback report distribution in 9 of the 13 months, although the surveys conducted in months 7 and 8 were combined, as they were conducted over 2 months across the four facilities, rather than surveys in each facility as were conducted during the other survey months. During those months, we were conducting an additional survey for a sub-study focused on social networks among staff in two of the four facilities [16] and needed additional time to complete the surveys in the other two facilities.

DICE-LTC had a total of 13 feedback months and eight survey cycles of post-feedback survey administration to obtain providers’ responses and reactions to the feedback reports, which is a longer duration with more extensive process evaluation than most audits with feedback interventions in the literature [3]. The timeline for the feedback report distribution and survey administration is shown in Additional file 1: Figure SA1–2. Some components of our process evaluation measurement, such as observation, occurred during feedback report distribution, although most occurred as part of the post-feedback survey. As the timeline indicates, we did not conduct surveys in every feedback month largely because of staffing constraints during summer months.

The purpose of the post-feedback survey was to assess staff response to the feedback reports, as well as to assess intention to change assessment of pain. The survey instrument is attached as Additional file 2. The process evaluation used the self-reported survey data and limited observation during feedback report distribution to assess uptake of the feedback reports. We were also interested in staff self-report of intention to change behavior following the intervention, which is an important intermediate outcome in the Theory of Planned Behavior.

### Settings and sample

The settings were nine LTC nursing units in four facilities in Edmonton, Alberta, Canada. The facilities had all implemented the Minimum Data Set/Resident Assessment Instrument version 2.0 (RAI 2.0) (http://www.interrai.org) at least 8–12 months prior to the beginning of the project. They ranged from one to three units providing care to LTC residents, for a total of nine nursing units, with between 40 and 75 beds in each unit. We included facility administrators, unit managers, and frontline direct-care staff, including registered nurses, licensed practical nurses, nurse aides (also called health care aides), physical therapists, recreational therapists, occupational therapists, pharmacists, social workers, and other allied health providers in our feedback report and survey distribution. We introduced the project to the staff at the four facilities through information sessions held over a month-long period before the intervention began, explaining our plans for distributing feedback reports, conducting surveys and observations, and the purpose of the project.

### Intervention methods

#### Feedback report distribution and behavior observation during feedback monthly periods

Feedback reports were developed through a pilot study involving two of the LTC facilities that were also included in DICE-LTC. The feedback reports used RAI 2.0 data from the participating units as the source data. We reported on measures of pain frequency and intensity, occurrence of falls, and depression prevalence, all aggregated to the unit level. We also reported on fall risk. Reports were primarily graphic with minimal text bullets, contained on one sheet of paper, front and back, printed in color. We also provided a cover sheet which explained key data issues to participants, such as how we calculated the pain, depression and falls risk scores and the period covered by the data and a brief statement about interpreting the report. An example is provided in the protocol paper [15]. The measures were based on nationally endorsed, validated Quality Indicators described in more detail in Additional file 3.

Data for the reports were lagged by about 6 weeks due to the time required to complete assessments and ensure that the data were available. Reports were hand-delivered by project staff in each of the nine nursing units during a consistent week during each of the 13 months of the intervention period. Each report was specific to the nursing unit, which we considered critical as the units were the basis of the teams providing care to residents, and care is organized on a unit basis. Two research assistants visited each unit at the same time to deliver reports. One research assistant observed staff behavior as they received reports and maintained counts of specific behaviors: whether the staff member read the report immediately or put it into his/her pocket instead of reading immediately, for example (observation form provided as Additional file 4).

#### Post-feedback surveys

We conducted surveys of all available direct-care staff in the four facilities. We made special efforts to maximize participation in the surveys during the first and last months of the intervention period to ensure that we had good baseline participation as well as good participation as the intervention ended. In all survey cycles, we provided treats for the staff, whether or not they completed the surveys, and offered $5 (Canadian) coffee gift cards to those who completed the surveys. In the first and last survey cycles (1 and 8), we also offered either breakfast or lunch/dinner for all staff, with decorations in the staff break rooms. Survey administration was a week after feedback report delivery, so the small incentives that we offered the staff for survey participation would not have affected their response to the feedback reports directly.

Surveys included questions to assess whether the staff read the reports and whether they considered them useful in their daily work to attempt to improve care to individual residents; if so, what kinds of actions were taken, and whether formal and less formal efforts at quality improvement were initiated. Surveys were anonymous, identifying only the nursing unit and facility where the staff member works and type of provider. We did this to improve response rates, but it had implications for our ability to track respondents over time, which we discuss later.

#### Process measures

We report findings for the following measures, noting whether the information is in the main text, or in Additional file 5:

- Survey response rates by facility and survey cycle (main text)
- Observation of what providers did when handed the feedback report, as well as number of reports left for later pickup by unit staff (main text)
- Proportions of respondents by facility and provider type reporting that they:
  - ○ Received the reports (Additional file 5)
  - ○ Read the reports (Additional file 5)
  - ○ Found the reports understandable (Additional file 5)
  - ○ Found the reports useful overall (Additional file 5)
  - ○ Discussed the reports with another staff member (main text)
    - ▪ What the discussion was about (Additional file 5)
    - ▪ Whether they discussed them in staff meetings during the last year (cycle 9 only) (Additional file 5)
- Found the reports useful to make changes in how they take care of residents (Additional file 5)

We included this group of process measures as measures of uptake of the feedback report in each feedback month. The measures build on each other: respondents needed to receive the report in order to read it, needed to read it to understand it, and needed at least to some extent to understand the report to find it useful or not. Discussing the report with another staff member follows our conceptual model and our expectation that the response to feedback in part will affect attitudes and perhaps social norms, but this is most likely if a participant discusses the report with another participant. Finally, the question of their perception of usefulness of the report to make changes in resident care is different from their perception of general usefulness of the report, which could be a less in-depth perception requiring less detailed thinking or processing.

Based on our understanding of uptake described above, we created a three-item scale to measure uptake defined as receiving, reading, and understanding by adding 1 to an uptake score for each individual when they:

- Reported that they had received the report during the feedback distribution the week before (0 otherwise)
- Reported that they had read more than half of the report (0 otherwise)
- And reported that they had understood more than half of the report (0 otherwise)

Changes in the questions about how much of the feedback report the respondent reported reading and understanding had an impact on the information we collected. In survey cycles 1 and 2, we asked dichotomous questions (“did you read the report?”), but in survey cycle 3 forward, we asked an ordinal question about how much of the report the respondent had read and understood. We made these changes because we realized after the first two survey cycles that a yes/no answer to these questions was not sufficiently informative about what respondents had done with the report, and that we needed more information to understand uptake.

#### Intermediate outcome measure: intent to change behavior—assessing pain in the next shift

We measured intention to change behavior using participant responses to the statement “I intend to assess residents’ level of pain during my next shift” on a 1 to 7 scale, with 1 = strongly disagree and 7 = strongly agree. We report the mean for each provider type, as well as the percentage of respondents by facility for the entire period (all eight survey cycles aggregated) who responded with at least a 4 or higher on the scale. We followed guidance on creating TPB surveys in creating this survey question [17]. We used this as the intermediate outcome described in the TPB, intention to change behavior, as a key indicator of participant response to the feedback intervention.

### Analysis

We provide descriptive summaries using both tables and graphs over survey cycles 1–9, over the four facilities. Because of the amount of data, we do not report unit-level data in this paper. We also provide graphs detailing response to specific survey items by provider (staff) type in three groups: registered nurses and licensed practical nurses (RN/LPNs), which include the unit care manager, HCAs, and Allied Health Professionals (AHPs). As noted above, we report much of the detailed findings in Additional file 5 because of the large volume of data.

We estimated the response rates as the number of responses from providers in a given time period divided by the number of resident beds over that time period. We attempted multiple methods of estimating response rates, all of which produced similar results (described in more detail in Additional file 1). To keep the tables simpler, we include survey cycles 1 and 9, corresponding to the beginning and end of our surveys, in reporting respondent demographics, aggregated in total and by facility. In reporting counts from observations, we include a total of 13 feedback months. In reporting provider responses to uptake questions on the survey instrument, we provide tabular data for survey cycles 1, 5, and 9, corresponding to the beginning, middle, and end of the survey administration periods, with graphic representation of responses to the same questions for all eight survey cycles to show trends over time. We show trends over time for the uptake scale overall, followed by trends over time for uptake by provider group and means by provider group for the intention to assess pain for residents on the next shift.

## Results

Our surveys, conducted over eight cycles, resulted in data from 1,080 participants over the entire intervention period. We provide detailed results from individual survey items over time and by provider group in Additional file 5 and focus our description of results here on findings pertinent to uptake, perceptions of usefulness, and discussion of the feedback report with other staff members, which all relate to the attitudes and beliefs, social norms, and perceived behavioral control dimensions of the TPB, as well as participants’ reports of their intentions to change their behavior and perform pain assessments with residents.

### Demographics and response rates

**Figure 1:**
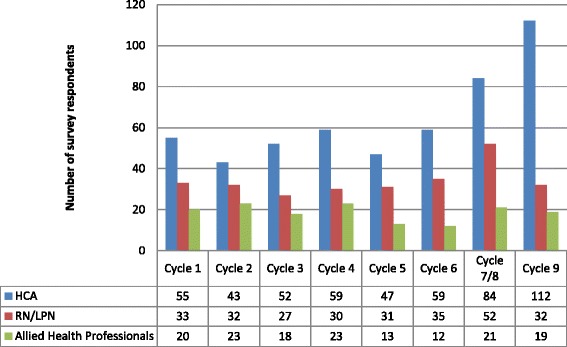
**Respondents by provider type and cycle.**

In the first survey cycle, at the beginning of the intervention, we had 126 respondents over the four facilities, ranging from 18 to 43 (Table 1). The largest number and proportion of providers were HCAs, numbering 59 across all four facilities, and ranging from 5 to 26 per facility. Other provider groups were considerably smaller, with small numbers of AHPs. These groups are in fact very small, with part-time and shared FTE distributed across both units and facilities. In the final survey cycle, at the end of the intervention, we had a total of 201 respondents, ranging from 15 to 72 across the four facilities (Table 2). Again, the largest single group was HCAs, with 110 respondents, ranging from 9 to 39 by facility. The years of experience either in long-term care or on the specific unit varied widely across facilities and also by survey cycle. This provides evidence that although we had some proportion of respondents who were the same across survey cycles, we had different respondents in different cycles. In Figure 1, we show the number of respondents by provider type across the eight survey cycles. In each time period, HCAs are the single largest group of providers, reflecting their numbers in LTC facilities, with varying numbers over time, and the largest numbers overall in the last two survey cycles.

**Table 1:** **Respondent demographics, survey cycle 1**

**Characteristics**	**Overall**		**Facility 1**		**Facility 2**		**Facility 3**		**Facility 4**	
	**Number**	**%**	**Number**	**%**	**Number**	**%**	**Number**	**%**	**Number**	**%**
Position title										
Care manager	3	2.4	−	−	2	5.9	1	2.3	−	−
Registered nurse	15	11.9	4	12.9	3	8.8	4	9.3	4	22.2
Licensed practical nurse	15	11.9	5	16.1	5	14.7	4	9.3	1	5.6
Health care aide	59	46.8	5	16.1	18	52.9	26	60.5	10	55.6
Social worker	1	0.8	−	−	−	−	1	2.3	−	−
Physical therapist/assistant	2	1.6	1	3.2	1	2.9	−	−	−	−
Recreational therapist/assistant	3	2.4	−	−	2	5.9	1	2.3	−	−
Occupational therapist/assistant	7	5.6	5	16.1	1	2.9	1	2.3	−	−
Pharmacist	3	2.4	1	3.2	−	−	−	−	2	11.1
Dietitian	1	0.8	1	3.2	−	−	−	−	−	−
Other	17	13.5	9	29.0	2	5.9	5	11.6	1	5.6
Total	126	100	31	100	34	100	43	100	18	100
Length of time working in long-term care (in years)										
Mean ± standard deviation	8.09 ± 8.05		13.30 ± 9.89		7.17 ± 7.37		6.42 ± 6.41		4.59 ± 4.82	
Median	5.00		12.00		4.00		4.00		3.00	
Length of time working on current unit (in years)										
Mean ± standard deviation	4.97 ± 5.30		6.39 ± 6.51		3.70 ± 3.59		5.24 ± 5.59		4.21 ± 4.57	
Median	3.00		3.00		2.75		3.00		3.00

**Table 2:** **Respondent demographics, survey cycle 9**

**Characteristics**	**Overall**		**Facility 1**		**Facility 2**		**Facility 3**		**Facility 4**	
	**Number**	**%**	**Number**	**%**	**Number**	**%**	**Number**	**%**	**Number**	**%**
Position title										
Care manager	3	1.5	−	−	3	5.3	−	−	−	−
Registered nurse	27	13.4	6	8.3	8	14.0	9	15.8	4	26.7
Licensed practical nurse	30	14.9	10	13.9	9	15.8	10	17.5	1	6.7
Health care aide/personal care attendant	110	54.7	39	54.2	26	45.6	36	63.2	9	60.0
Social worker	1	0.5	−	−	1	1.8	−	−	−	−
Physical therapist/assistant	12	6.0	6	8.3	6	10.5	−	−	−	−
Recreational therapist/assistant	2	1.0	2	2.8	−	−	−	−	−	−
Occupational therapist/assistant	1	0.5	−	−	1	1.8	−	−	−	−
Pharmacist	1	0.5	1	1.4	−	−	−	−	−	−
Dietitian	−	−	−	−	−	−	−	−	−	−
Other	14	7.0	8	11.1	3	5.3	2	3.5	1	6.7
Total	201	100	72	100	57	100	57	100	15	100
Length of time working in long-term care (in years)										
Mean ± standard deviation	11.26 ± 9.14		13.57 ± 10.19		10.80 ± 9.34		10.14 ± 7.56		6.50 ± 5.86	
Median	9.00		12.00		8.50		9.00		4.50	
Length of time working on current unit (in years)										
Mean ± standard deviation	5.70 ± 5.25		6.43 ± 5.96		4.44 ± 4.67		6.78 ± 4.83		3.74 ± 4.15	
Median	4.00		4.00		3.00		5.50		1.75

**Figure 2:**
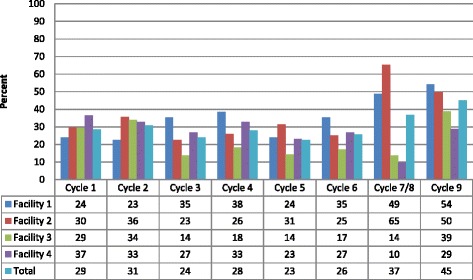
**Survey response rates based on bed counts over the nine survey cycles.**

In Figure 2, we show the numbers and rates for survey completion across all four facilities and the eight survey time cycles. Our lowest response rate was in cycle 5 (23%), in the middle of the intervention period, and our highest was at the end of the intervention period (45%). Survey response rates varied across the intervention period and corresponded in part to issues related to conditions in the facilities. An example of facility conditions that affected our data collection included an outbreak of a gastrointestinal virus in one facility that forced us to leave feedback reports and surveys for internal distribution because our staff were not allowed into the facility that month.

### Behaviors observed during feedback report distribution

We observed a total of 2,365 behaviors across the 13 feedback months, ranging from 319 in the smallest facility to 759 in the largest. The vast majority were “Report not given directly to an individual”. Staff members were frequently observed to read the report, sometimes asking the RA questions about it. The staff were often observed putting the report in their pocket or somewhere else; this occurred 7%–23% of the time. The staff were only infrequently observed discussing reports with other staff, although the period of observation following report distribution was brief and would not have captured later conversations. In a few cases, the staff refused the reports, and in only one case, a staff member was observed throwing the report into the trash after receiving it.

**Figure 3:**
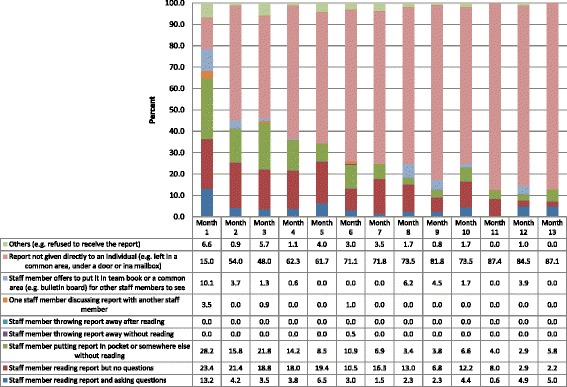
**Provider behaviors in feedback report distribution over the 13 feedback months.**

Over the 13 feedback months (Figure 3), the peak of staff reading the report and asking questions was in the first distribution cycle (13% of all observed behavior), although the two most common behaviors in that cycle were reading the report without asking questions (23%) and putting the report into a pocket or somewhere else (28%). In feedback month 1, only 15% of feedback reports were left for later distribution or pickup, rather than handed to an individual staff person, whereas in feedback month 13, 87% were left for later distribution. This shift to leaving reports for staff to pick up later was largely due to increasing familiarity with project RAs, and staff feeling too busy to spend time with them as they distributed the report.

### Provider response to feedback reports across facilities

We obtained 1080 responses to the post-feedback surveys across all eight Survey Cycles and all four facilities (Tables 3 and 4). Overall, 79% of respondents said they had read the feedback reports. The range across the four facilities was 76%–88%. Most respondents (78%) said that they found the reports understandable (facility range 78%–83%). A smaller proportion (64%) said they found the reports useful in general (54%–73%), while 66% reported that they found the information in the reports useful to make changes in how they take care of residents (65%–72%). Overall, 42% reported discussing the feedback reports with another provider (41%–48%). We discuss these findings further as we discuss the uptake scale calculated from these data.

**Table 3:** **Provider responses to post-feedback survey items by facility over entire intervention period**

**Provider responses to feedback reports**	**Overall**		**Facility 1**		**Facility 2**		**Facility 3**		**Facility 4**	
	**(** ***N*** **= 1,080)**		**(** ***N*** **= 376)**		**(** ***N*** **= 328)**		**(** ***N*** **= 263)**		**(** ***N*** **= 113)**	
	**Number**	**%**	**Number**	**%**	**Number**	**%**	**Number**	**%**	**Number**	**%**
Proportion of respondents who state they have read the report	858	79.4%	290	77.1%	255	77.7%	215	81.7%	98	86.7%
Proportion of respondents who find the report understandable	841	77.9%	291	77.4%	248	75.6%	215	81.7%	87	77.0%
Proportion of respondents who find the report generally useful	689	63.8%	269	71.5%	174	53.0%	180	68.4%	66	58.4%
Proportion of respondents who discussed the report with another staff member	455	42.1%	152	40.4%	137	41.8%	112	42.6%	54	47.8%
Proportion of respondents who find the report useful to make changes in the way they take care of residents	710	65.7%	251	66.8%	205	62.5%	182	69.2%	72	63.7%

**Table 4:** **Proportion of survey respondents who report that they intend to assess pain during the next shift over the entire intervention period**

**Facility**	**Number of respondents over entire intervention period**	**Proportion who report they intend to assess pain**
F1	291	83%
F2	261	87%
F3	215	88%
F4	97	92%
Overall	874	86%

Numbers differ from previous tables as only direct care providers were included in this section of the survey.

### Uptake scale findings

**Figure 4:**
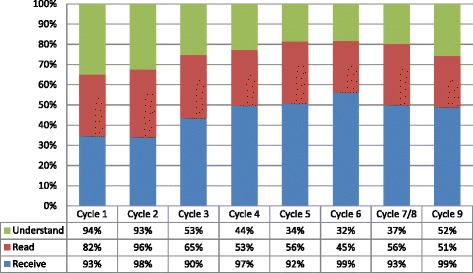
**Uptake scale for all participants cycles 3–9.**

Overall, across all provider types, the results for the uptake scale were high across cycles 1 through 9 (Figure 4). Receipt of the report increased over the period, while the proportion of all providers stating that they had read or understood the report varied by survey cycle. It is important to note that the change in the questions about reading and understanding the report from binary to ordinal affected the proportion of providers scored as reading or understanding the report, particularly between cycles 1 and 2. Uptake scale findings varied by provider type over the period (Additional file 5). The scores were not monotonic for any provider group but varied by survey cycle. Receipt of the surveys was high, but reading and understanding varied considerably by cycle. The changes observed by group reflected not only different compositions of the staff during the cycles but also staff workload and relative scarcity over time. We present much more detail about each of the elements of the uptake scale (receiving, reading, and understanding) as well as detail about provider perceptions of overall usefulness in Additional file 5.

### Discussing the report with another staff member

**Figure 5:**
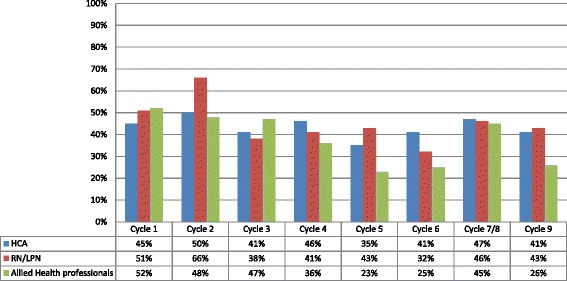
**Respondents who report discussing the feedback report with another staff member by provider type and cycle.**

We focused on discussions of the feedback reports among the staff because of our conceptual model, which posited that one important mechanism of feedback action would be through possible changes in attitudes and beliefs brought about by discussion among the staff. In the TPB, there are interactions among the three mechanisms that affect the intention to change behavior (Additional file 1), and social norms can influence attitudes and perceived behavioral control. In Figure 5, reports from participants that they discussed the feedback reports with other staff were highest in the first two survey cycles, across all provider types. After survey cycle 2, proportions of HCA and RN/LPN respondents who reported discussing feedback reports with other staff dropped off, while AHP proportions stayed high until after survey cycle 3. In cycles 5 and 6, reports of discussion among the staff are low for all provider types, particularly AHPs, and then there is an increase again in reports of discussion in cycles 7/8 and 9. There is not a clear, consistent pattern of discussion.

### Intention to change behavior—assess pain in the next shift

In the TPB, the intention to change behavior is an important intermediate outcome that predicts actual behavior change. We measured this for pain assessment only among the four elements of the feedback report. Overall, across the entire intervention period, the proportion of respondents who said that they intended to assess pain among the residents they cared for in the next shift was high, with slight variation by facility (Table 4). Facility 1, the largest of the four facilities, had the lowest proportion reporting a positive intention over the entire period (83%), while facility 4, the smallest of the facilities in the study, reported the highest (92%).

**Figure 6:**
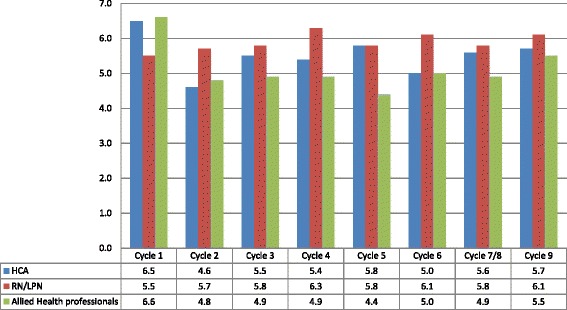
**Means reported on 1 to 7 scale for the intention to assess pain on next shift by provider type and cycle.**

We show mean responses to the 1 to 7 scale asking how strongly respondents disagreed (1) or agreed (7) to the statement “I will assess pain among the residents I care for on my next shift” by provider type and cycle in Figure 6. Mean responses were relatively high overall, with the lowest mean (4.4 out of 7) reported for AHPs in cycle 5 and the highest (6.6) for AHPs in cycle 1. Across all nine cycles, RNs and LPNs reported the highest means, while AHPs reported the lowest. Except for cycle 1, when HCAs reported the highest mean for the three provider types (6.5), their means were lower than RNs and LPNs, but generally higher than AHPs. These differences may reflect different roles and responsibilities for pain assessment in this setting.

## Discussion

This paper is the first, to our knowledge, to present in-depth, longitudinal process data about an intensive feedback report intervention over an extended period of time, as well as reporting an intermediate outcome such as intention to change behavior. In doing so, we are responding to calls for improved reporting of interventions, providing enough information for readers and future researchers in this area to assess how to conduct the intervention, as well as its effects [18-20]. Our findings show that uptake is not easily captured by any single dimension, and that it was not monotonic in either direction over the period of the intervention. However, measured by the uptake scale that summarizes three key factors related to participants’ responses to the feedback report, uptake appears to have been relatively high across the intervention period.

This raises the question of why the feedback intervention was less successful than we had expected as we initiated the study, based on the most current reviews of feedback interventions [21,22]. As we discuss in the summative report of the intervention [6], low staffing levels and lack of other resources in these settings probably contributed to the lack of ability to take time to read even a short, graphic feedback report. In addition, the level of hierarchy among the staff providing care, with HCAs clearly at the bottom of the hierarchy with little ability to enact decisions or influence policy, may also have contributed to the lack of action in response to the feedback reports. As shown in Additional file 5: Figure S5–10, HCAs most consistently reported that they found the reports useful in changing resident care, but they have the least scope of practice and scope of control over resident care practices. This may appear anomalous, but this anomaly has been shown in previous studies that include HCAs in long-term care and other settings [23], and may be related to persistent optimism necessary to remain engaged in caring despite lack of control over the work environment.

It is possible that providing additional feedback through verbal reports, rather than written alone, may have improved both uptake and effectiveness. There is evidence that written reports are more effective than verbal [3,22], and delivering the reports verbally in a group setting may have made it more likely that staff members would have discussed the reports with each other. However, there are also some potential problems with this approach, particularly in LTC settings. First, there is evidence that when feedback recipients feel both uncertainty and a lack of psychological safety, they are likely not only not to act on the reports, but under these circumstances, the reports can actually lead to taking action in an undesired direction [24,25], even leading to actions not anticipated or desired. In the low resource, high hierarchy world of long-term care, this would not be surprising. More pragmatically, finding time to deliver verbal reports would have been almost impossible in this setting, which leads back to the reality that time and staffing constraints limit the effect of any intervention that requires staff time.

Our study is unique in many respects. First, the vast majority of feedback interventions reported in the peer-reviewed literature target a single, relatively homogenous group of providers, typically licensed professionals, and most, to date, have targeted physicians [3]. Our study is one of the first to target a broad range of staff, ranging from highly professionalized care managers (most of whom are registered nurses) and allied health professionals to health care aides. This gives us a glimpse into the full range of health care providers who constitute the care team in LTC settings and their responses to feedback reports.

Second, we hand-delivered reports to staff, an unusual method of feedback intervention delivery. This offered a number of important advantages: we were able to observe staff behavior at the point of report receipt, we have a better idea than in many studies of how many people received the report, we offered staff an opportunity to engage by asking questions, and we demonstrated our commitment to the intervention by our presence and attention. Our delivery method was, however, dependent on factors within each unit in different months. Low points in staffing, particularly during the summer months (months 5–8, cycles 5, 6, and 7/8), diminished the staff capacity to interact and engage. To some extent, issues like these are inevitable in a long prospective intervention study. A major advantage of ongoing, consistent process evaluation is our ability to observe some of these effects.

Third, we provided reports to individuals who were members of care teams that largely, in LTC settings, operate at the level of a nursing unit. Despite providing the reports to individuals, the information in the report was at the unit level. This is different from many feedback interventions, where the level of distribution (team/individual) is the same as the level of the information in the report (team/individual performance). We argue that in nursing care, performance is always through teams rather than individuals, so that measuring at the team (in this case, unit) level makes the most sense. However, ensuring that all members of the team receive the reports is important if all individuals are expected to make changes to improve care. We did not vary this factor in our study, so we do not have data showing the effect of providing reports to team vs. individuals when the report measures performance at the team level, but this would be an important design feature to test in future studies [26].

Given the relatively high uptake scale scores, the overall lack of effectiveness of the feedback intervention was likely not due to inadequate uptake of the reports [6]. However, the amount of positive endorsement of each of these elements of uptake varied over the period of the intervention and by provider type, and we have evidence that how often staff discussed the feedback reports with each other—related to social norms and their effect on attitudes—was not very frequent overall, and the degree to which staff discussed ideas for resident care based on the feedback reports was low at the end of the intervention period compared to the beginning. This suggests that adding a co-intervention that helps stimulate staff discussion and thinking about ways in which to apply the report to resident care might help increase the effectiveness of a feedback intervention in these settings. Overall, only a small amount of time is available for education and staff meetings. In addition, it is important to note that while the intervention overall showed little effect, there was a small and significant improvement in the proportion of residents with moderate or severe pain. Intention to change pain assessment behavior was the focus of the TPB section of the post-feedback questionnaire, and it may have acted as a co-intervention as we suggest, by focusing staff attention on the issue and providing clear suggestions about one step to take to better manage pain, by increasing pain assessment.

We expected that the feedback intervention would operate at least partially through social influences, specifically staff members talking to each other about the reports. Our findings suggest that although staff did discuss the reports with each other, these discussions were not widespread. This may be due to social structure within the units, which is often hierarchical and tightly bound by type of provider, especially in long-term care settings [27]. We found variation in discussion by provider type and cycle. Discussion among all provider types was lowest during the high workload summer months and highest at the beginning of the intervention, when it was novel. The AHP group had the greatest decrease in discussing the reports, perhaps partly because they are few in number and susceptible to workload stress, but also because they found the reports less interesting to them as time went on. However, AHPs were most likely to report discussing the feedback reports in staff meetings, which may have led to less individual discussion with another staff member. Advocating for discussion in the staff meetings that are available may be another method of increasing not only staff attention to the feedback reports but also a way of stimulating the exchange of ideas and perspectives that is likely critical to changing attitudes and social norms.

Providers saying that they found the feedback reports useful to change resident care is a key indicator of uptake beyond receiving, reading, and understanding (included in the uptake scale). As a mechanism for changing behavior, finding useful information in the reports specific to changing resident care (as opposed to general usefulness, reported in Additional file 5: Figure S5–5) is likely to support new behavior by enhancing specific knowledge. As with approaches to changing provider attitudes and social norms, providing useful information that supports new behavior may be an important factor to consider in designing feedback interventions.

The reported intention to assess pain among residents on the next shift was high from the outset and stayed relatively high throughout the intervention period. However, there were differences by provider type, some of which may have been related to roles and functions. RNs and LPNs have the greatest responsibility for assessing pain, in most LTC settings, but often have little direct contact with residents. HCAs, who have the greatest resident contact, are often discouraged from activities like “assessment”, which is sometimes thought to be reserved to licensed professionals. We had several discussions about this during the course of the project, among both clinician researchers, and facility and organizational leaders. It is clearly a point of tension. Despite this, HCA survey respondents generally reported relatively high mean intention to assess pain, slightly lower than RN and LPN respondent means, but higher than AHPs. The heterogeneity among AHPs in their roles and training may be one reason they had lower means. Not all AHPs participating in the survey would consider pain assessment to be within their scope of practice (for example, recreation therapists, social workers, or dietitians).

### Limitations

While we attempted a comprehensive process evaluation, we did not obtain in-depth qualitative data about staff thoughts and experiences as they received, read, discussed, and assimilated, and perhaps acted on, the feedback reports. This level of engagement was beyond the scope of the project, and although we believe that our findings offer important insights for further research, in future work, we would attempt to build in more opportunity for qualitative data collection.

We were limited to a small number of facilities (four) and nursing units (nine). All of the facilities and units were in a single urban area. While Edmonton shares characteristics with medium-sized cities elsewhere in North America, it also has some unique characteristics (very rapid population growth over the last several decades; high economic activity with very tight labor markets over the period of the study, compared with slumping economies and relatively high unemployment in other markets; rapid influx of immigrants over the last two decades) that may restrict the generalizability of our findings.

The relatively low survey response rates in our study—calculated conservatively by using number of beds as the denominator—are a limitation of the study but are also common for projects in LTC settings. We worked intensively to maximize response, by covering all three shifts and offering small incentives for participation, including refreshments. Our response rates are consistent with those in other studies in these resource and personnel-constrained settings.

The labor-intensive process of generating the reports presented a barrier to our intervention. We were able to automate the actual generation of the graphs in the report, but not the bulleted text. The major work lies in obtaining and cleaning the data to have sufficiently consistent and accurate data for the reports. We were not able process the data without a minimum 6-week lag. This was in part due to staffing limitations in our research team, more importantly due to the lag in closing data in each assessment period. This highlights the importance of understanding the nature of the data stream being used to produce feedback reports. While using electronic data from clinical practice and resident care planning is a major strength, it also imposes constraints and limitations. In addition to the problems with time-lagged data, which were commented on by some participants, the monthly generation and distribution of reports was very time- and energy-consuming. In part because of this, we were not able to use our process data in a formative mode, in which we might have made changes to the intervention in response to issues identified in the process data.

### Implications for decision-makers and researchers

We have demonstrated that a relatively intensive feedback intervention can be carried out in LTC settings with high uptake and staff participation, despite the lack of resources and low levels of staffing compared to other health care settings. However, challenges in maintaining the intervention, how best to supplement it with targeted education or other behavior change techniques [26,28] and how to deliver it in a fully cost-effective approach, still remain. However, data systems are evolving rapidly in Alberta and elsewhere, and the considerable attention to quality of care in LTC settings is a strong asset in maintaining and enhancing this work [2,16].

There are still many questions about how best to design and deliver feedback interventions, as well as how best to conduct process evaluations during the course of feedback interventions. Syntheses and critical appraisals of the feedback intervention literature have pointed out significant gaps in the reports of feedback interventions, some of which may be due to limitations in publication (word limits, for example), while others are due to limited planning and conceptualization of how to conduct a feedback intervention [18,21,28,29]. We argue that simply reporting the effectiveness of a feedback intervention is insufficient to answer how and why the investigators found the effect they did, although it may answer the question of whether that specific intervention was effective or not. Reporting on uptake of the intervention is essential, and measuring key factors that might contribute to effectiveness and success of implementation is also very important.

## Conclusions

Our paper provides details of key process points and uptake metrics in a longitudinal quasi-experimental study of a feedback intervention in LTC settings. We find variation over time and place in key measures of uptake and staff receptivity to a 13-month feedback intervention, although we also document a relatively high level of engagement over a long period of time on the part of staff in a resource-constrained environment.

## Additional files

Additional file 1:
**Design and methods.**
Additional file 2:
**Data for improvement and clinical excellence (DICE) post-feedback report survey.**
Additional file 3:
**Overview of Proposed new CCRS Quality Indicators (see attached for detailed description of parameters).**
Additional file 4:
**Observational checklist.**
Additional file 5:
**Additional results and findings.**
