# Supplementary material for: Understanding feedback report uptake: process evaluation findings from a 13-month feedback intervention in long-term care settings
Source: Implement Sci. 2015 Feb 12;10:20. doi: 10.1186/s13012-015-0208-2 (PMC4331147; doi:10.1186/s13012-015-0208-2)
Supplement: Additional file 1: — Design and methods. [file 13012_2015_208_MOESM1_ESM.docx]

**Design and Methods**

We conducted this intervention project, called the Data for Improvement and Clinical Excellence in Long Term Care (DICE-LTC), over a thirteen month period in nine long term care (LTC) units in four facilities. The overall intervention involved monthly feedback reports distributed in nine long term care units in four facilities. We conducted surveys to assess uptake of the audit with feedback intervention one week after feedback report distribution in most, but not all months. The purpose of the survey was to assess staff response to the feedback reports, as well as to assess intention to change assessment of pain. The survey instrument is attached as an appendix (Additional File 2). Our focus in this paper is on Sections A and B of the survey, reporting on brief demographic information as well as information about response to the feedback report, although we report descriptive findings related to intention to change behavior.

The process evaluation, conducted concurrently with the feedback report distribution, used the survey data, observation and self-report to assess uptake of the feedback reports. We define uptake as receiving, reading, and understanding the feedback reports, as well as discussing with colleagues and managers. We were also interested in their report of intention to change behavior based on the reports. The overall methods of the study were described in detail in a protocol paper[1].

The conceptual model for the study was based on the Theory of Planned Behavior, shown in Figure A1-1 in this document, and described in the main text.

*The intervention*

DICE-LTC had a total of 13 Feedback Months and 8 Survey Cycles of post-feedback survey administration to obtain providers’ responses and reactions to the feedback reports (Figure A1-2 in this document). Some components of our process evaluation measurement, such as observation, occurred during the feedback report distribution, although most occurred as part of the post-feedback survey conducted a week after distribution of the surveys. We did not conduct post-feedback surveys in each Feedback Month, due to staff shortages in the summer months. We combined Cycles 7 and 8, with each facility only receiving surveys once during this two-month period. We conducted a sub-study focusing on social networks related to the feedback reports including only two of the four facilities[2], necessitating additional data collection in those two facilities, and altering our pattern of data collection in the other two facilities as a result. As a result of these modifications, we had a total of 8 Survey Cycles rather than 13.

**Settings and sample**

The settings were nine long term care nursing units in four facilities or nursing homes in Edmonton, Alberta, Canada. The facilities had all implemented the Minimum Data Set/Resident Assessment Instrument version 2.0 (RAI 2.0) (<http://www.interrai.org>) at least 8-12 months prior to the beginning of the project. The four facilities were part of two separate organizations providing long term care and other residential services in Edmonton. They ranged from one to three units providing care to LTC residents, for a total of nine nursing units, with between 40 and 75 beds in each unit. The four facilities were part of two organizations providing a range of continuing care services in the community. All four facilities had implemented the Resident Assessment Instrument Minimum Data Set (RAI) version 2.0 (<http://www.interrai.org>), although two facilities had implemented RAI 2.0 between 3 and 5 years prior to the beginning of the DICE-LTC feedback intervention, while the other two had only implemented this system between 6 and 12 months prior to the project beginning. The organizations were recruited through their participation in the Knowledge Brokering Group, which had begun as a project funded by the Canadian Health Services Research Foundation to support long term care organizations interested in using data for quality assessment and quality improvement. We included all facility-based staff in our feedback report intervention and survey distribution.

Facility directors received the reports a week before they were distributed, so that they were aware of the data being provided to staff, and also as a check on accuracy. Only once did we make any changes to a feedback report during the intervention period, when a director pointed out an error in the report. Administrators were not involved in survey administration, and did not have access to completed surveys. We did not report to them about completion rates, or other information related to the conduct or findings of the survey during the project, although some senior leaders within the two organizations were members of the research team, and did get progress reports on preliminary and final study findings, in aggregate form only. Team members did not request data specific to each facility.

# Methods

Feedback report distribution and behavior observation during Feedback Monthly periods

Feedback reports were developed through a pilot study involving two of the LTC facilities that were also included in DICE-LTC. In the pilot study, we asked all direct-care providers to rate the importance, for quality improvement, of the resident functional status and outcome domains available in the RAI 2.0 data. After discussion with organizational leaders participating on the project team, the top-rated domains were developed into prototype feedback reports, using data from the facilities, and piloted with all direct-care staff in both facilities. The pilot study was completed before DICE-LTC began. The four items reported in the feedback report were the areas rated as most important to improve the quality of care by staff in the two facilities that participated in the pilot study: proportion of residents on each unit who reported high pain scores, proportion of residents with high depression screening scores, proportion of residents at risk of falls, and rate of falls on each unit in the previous 90 to 180 days.

The feedback reports used RAI 2.0 data from the participating units as the source data. With the exception of fall risk and depression, for which there is no standardized indicator, the indicators on the report were calculated based on quality indicators developed by the InterRAI Consortium and the Canadian Institute for Health Information ([www.cihi.ca](http://www.cihi.ca)). Our goal was to provide the units with data showing information about resident outcomes using indicators that are widely used in Canada (see Additional File 3 for more information on the quality indicators in effect at the time of the project). While these indicators were not reported publicly or otherwise in Alberta during the period of our project, there were plans to create public reports in the future.

Reports were primarily graphic with minimal text bullets, contained on one sheet of paper front and back, printed in color. We also provided a cover sheet which explained key data issues to participants. An example is provided as an appendix in the protocol paper[3].

Data for the reports were lagged by about 6 weeks. We received the data for each month approximately 10 days after the end of each month. With the exception of the final Feedback Month, we reported monthly data. We needed at least two weeks to process the data, and one week to allow facility directors to review the monthly report prior to distributing the data. During the final Feedback Month (FM 13), we changed the reports to reflect quarterly time points rather than monthly. This was partly done because we were anxious to provide reports that could be generated by the facilities themselves after the research intervention ended, and quarterly feedback reports were easier to prepare. We also found that the data generating monthly reports were not very stable.

Reports were hand delivered by project staff in each of the nine nursing units during a consistent week during each of the 13 months of the intervention period (Months 1-13). Each report was specific to the nursing unit. All direct care providers in all disciplines who were available for hand distribution, and managers in each unit, received the unit-specific reports. Hand delivery was accomplished by a research assistant visiting the unit, who handed feedback reports directly to providers who were working at the time of delivery. Reports were put into mailboxes or left in break rooms for providers not working during delivery cycles. Two research assistants visited each unit at the same time to deliver reports. One research assistant observed staff behavior as they received reports, and maintained counts of specific behaviors: whether the staff member read the report immediately, or put it into his/her pocket instead of reading immediately, for example (observation form provided as Additional File 4). Research assistants received on the job training from the project manager. All had at least bachelor’s degrees, and many had master’s degrees in varying fields, mostly in the sciences. RAs visited the facilities during multiple shifts in each week when feedback reports were distributed in that facility, to try to maximize the number of individual providers to whom feedback reports were given.

Post-feedback surveys

As with feedback report distribution, RAs visited the facility scheduled each week during multiple shifts to try to maximize response rates. We tried to cover night and day shift by visiting during early morning hours, and day and evening shift by visiting during late afternoon hours. We made special efforts to maximize participation in the surveys during the first and last months of the intervention period.

Surveys included questions to assess whether staff read the reports, whether they considered them useful in their daily work to attempt to improve care to individual residents; if so, what kinds of actions were taken, and whether formal and less formal efforts at quality improvement were initiated. The first question asking whether the staff member had read the report was defined as meaning that they had read the report prior to survey administration; if they had not read it prior to survey administration, they were offered a report to read so that they could respond to other questions on the survey. These questions addressed issues of feedback report uptake. Surveys were anonymous, identifying only nursing unit and facility where the staff member works, and type of provider. We made this decision as part of our effort to increase the likelihood of staff response. This had implications for our ability to track respondents over time, which we discuss later.

Process measures

We report on process findings, specifically the following:

- Survey response rates by facility and month
- Observation of what providers did when handed the feedback report, as well as number of reports left for later pick-up by unit staff
- Proportions of respondents by facility and provider type reporting that they:
  - Received the reports
  - Read the reports
  - Found the reports understandable
  - Found the reports useful overall
  - Discussed the reports with another staff member
    - What the discussion was about
    - Did they discuss in staff meeting during the last year (Cycle 9 only)
  - Found the reports useful to make changes in how they take care of residents.

We included this group of process measures as measures of uptake of the feedback report in each Feedback Month. In a sense, each measure builds on the previous one, at least among the first four items. Respondents needed to receive the report in order to read it, needed to read it to understand it, and needed at least to some extent to understand the report to find it useful or not. Discussing the report with another staff member follows our conceptual model and our expectation that the response to feedback in part will affect attitudes and perhaps social norms, but this is most likely if a participant discusses the report with another participant. Finally, the question of their perception of usefulness of the report to make changes in resident care is different from their perception of general usefulness of the report, which could be a less in-depth perception requiring less detailed thinking or processing.

Changes in the post-feedback survey affected responses to several of these questions. In the first version of the survey, used in Cycles 1 and 2, we asked binary questions (yes/no) about whether or not the respondent had read or understood the survey. From Cycle 3 forward, we asked these two as ordinal questions with four categories of response to each (less than half, half, more than half, all), which made a change in the level of the responses. In Figure 5, showing overall responses over the entire intervention period, we show the proportion of respondents who said they read or understood at least half of the report. In Figures 8 and 9, showing responses by provider type and cycle, for both the question about how much they read and how much they understood the report, we present the proportion of respondents who read or understood all of the feedback report, assuming this represents the proportion most interested in the feedback. For the question about how generally useful they found the report, we present the proportion who said that they considered the report at least “somewhat useful”. The two final questions, whether they discussed the report with another staff member, and whether they considered the report useful to change the way they take care of residents, were binary (yes/no) questions, and we report the proportion of respondents who responded affirmatively to each question.

We also provide more detail about what the survey respondents said that they discussed with another staff member. We asked respondents who indicated that they discussed the report whether they were discussing it with another staff member to get their thoughts about the report, and/or whether they were asking for advice for how to improve care for residents based on the report. They were also given the option of describing other reasons for discussing the report, and we captured brief statements about these other reasons. Finally, we asked respondents in Cycle 9 only, at the end of the intervention period, to report whether or not they had discussed the feedback report during a staff meeting in the last year, and if so, how often.

Intermediate outcome measure: intent to change behavior

We measured this using participant responses to the statement “I intend to assess residents’ level of pain during each shift” on a 1 to 7 scale, with 1=strongly disagree and 7 =strongly agree. We report the mean for each provider type, as well as the percentage of respondents by facility for the entire period (all nine Survey Cycles aggregated) who responded with at least a 4 or higher on the scale. We followed guidance on creating TPB surveys in creating this survey question[4].

**Analysis**

We provide descriptive summaries using both tables and graphs over Survey Cycles 1-9, over the four facilities. For tractability, we do not report unit-level data in this paper. We also provide graphs detailing response to specific survey items by provider type in three groups: registered nurses and licensed practical nurses (RN/LPNs), which include the unit care manager; health care aides (HCAs); and Allied Health Professionals (AHPs), including occupational, recreational, and physical therapists, and their aides, as well as pharmacists and social workers in some instances. The mix of AHPs varied by Survey Cycle.

We estimated response rates as the number of responses from providers in a given time period divided by the number of resident beds over that time period. We attempted multiple methods of estimating response rates, none of which were very successful. The facilities were not able to give us accurate counts of who worked on a unit or in a facility on any given day. While our RAs made an effort to count the number of providers they saw as they did distributions, these were not very accurate, although they form the basis for the observation count data we report. The facilities did not keep regular records of who worked on each unit after shifts were completed, and payroll data did not provide any information about where someone had worked. As noted previously, we were often asked to leave both feedback reports and surveys in the units or facilities, usually in a break room, for staff members not on site during feedback report distribution and/or survey administration to read or complete. While we tried to assess how many of these were actually taken by unique individuals, these efforts were generally not successful. As a result, we treat our analyses in this paper as descriptive, not inferential.

To keep the tables simpler, we include Survey Cycles 1 and 9, corresponding to the beginning and end of our surveys, in reporting respondent demographics, aggregated in total and by facility. In reporting counts from observations, we include a total of 13 Feedback Months. In reporting provider responses to uptake questions on the survey instrument, we provide tabular data for Survey Cycles 1, 5, and 9, corresponding to the beginning, middle and end of the survey administration periods, with graphic representation of responses to the same questions for all 9 Survey Cycles to show trends over time.

We did not track respondents over the survey cycles because we guaranteed them anonymity. We have attempted to link survey responses retrospectively using demographic information from the surveys without having knowledge of the individuals, and we found that less than 30% of survey responses were from the same individuals over the last three survey cycles. This suggests that if we were to link from the beginning to the end of the intervention period, only a small proportion of respondents would have participated over the entire period. However, many respondents responded multiple times over the entire intervention period.

| **Activity** | September 2007-December 2008 | November-December 2008 | Jan-09 | Feb-09 | Mar-09 | Apr-09 | May-09 | Jun-09 | Jul-09 | Aug-09 | Sep-09 | Oct-09 | Nov-09 | Dec-09 | Jan-10 | February- July 2010 |
| --- | --- | --- | --- | --- | --- | --- | --- | --- | --- | --- | --- | --- | --- | --- | --- | --- |
| **Pre-intervention data extraction** |  |  |  |  |  |  |  |  |  |  |  |  |  |  |  |  |
| **Baseline context assessment** |  |  |  |  |  |  |  |  |  |  |  |  |  |  |  |  |
| **Feedback report distribution** |  |  | Month 1 | Month 2 | Month 3 | Month 4 | Month 5 | Month 6 | Month 7 | Month 8 | Month 9 | Month 10 | Month 11 | Month 12 | Month 13 |  |
| **Survey administration** |  |  | Cycle 1 | Cycle 2 | Cycle 3 | Cycle 4 |  | Cycle 5 |  |  | Cycle 6 | Cycle 7/8 | |  | Cycle 9 |  |
| **Post-intervention data extraction** |  |  |  |  |  |  |  |  |  |  |  |  |  |  |  |  |
|  |  |  | **Intervention Period** | | | | | | | | | | | | |  |

Figure 2: Timeline for DICE-LTC overall
