# Supplementary material for: Understanding feedback report uptake: process evaluation findings from a 13-month feedback intervention in long-term care settings
Source: Implement Sci. 2015 Feb 12;10:20. doi: 10.1186/s13012-015-0208-2 (PMC4331147; doi:10.1186/s13012-015-0208-2)
Supplement: Additional file 3: — Overview of Proposed new CCRS Quality Indicators (see attached for detailed description of parameters). [file 13012_2015_208_MOESM3_ESM.doc]

**Overview of Proposed new CCRS Quality Indicators**

**(see attached for detailed description of parameters)**

| **Quality domain** | **#** | **Code** | **Quality Indicator** |
| --- | --- | --- | --- |
| ADL | 1 | ADL01 | Percent of residents who had an unexpected loss of function in some basic daily activities |
|  | 2 | ADL05 | Percent of residents who improve status on mid-loss ADL functioning (transfer, locomotion) or remain completely independent in mid-loss ADLs |
|  | 3 | ADL06 | Percent of residents who improve status on early-loss ADL functioning (dressing and personal hygiene) or remain completely independent in early-loss ADLs (ELADL). |
|  | 4 | ADL1A | Percent of residents who had an improvement of function in some basic daily activities |
|  | 5 | ADL5A | Percent of residents who declined status on mid-loss ADL functioning (transfer, locomotion) or remain completely dependent in mid-loss ADLs |
|  | 6 | ADL6A | Percent of residents who declined status on early-loss ADL functioning (dressing and personal hygiene) or remain completely dependent in early-loss ADLs (ELADL). |
|  | 7 | ADL7D | Proportion of ADLs changes that are decline |
| Behaviour | 8 | BEHD4 | Percent of residents who have declining behavioral symptoms |
| Continence | 9 | CAT02 | Percent of residents with indwelling catheters |
|  | 10 | CNT02 | Percent of residents with worsening bowel continence |
|  | 11 | CNT03 | Percent of residents with worsening bladder continence |
|  | 12 | CNT04 | Percent of residents with a urinary tract infection |
|  | 13 | CNT2A | Percent of residents with improving bowel continence |
|  | 14 | CNT3A | Percent of residents with improving bladder continence |
| Cognitive function | 15 | COG01 | Percent of residents whose cognitive ability has worsened |
|  | 16 | COG1A | Percent of residents whose cognitive ability has improved |
| Communication | 17 | COM01 | Percent of residents whose ability to communicate has worsened |
|  | 18 | COM1A | Percent of residents whose ability to communicate has improved |
| Delirium | 19 | DELOX | Percent of residents with symptoms of delirium |
| Falls | 20 | FAL02 | Percent of residents who have fallen in the last 30-days |
| Infection | 21 | INF0X | Percent of residents with infections |
|  | 22 | RSPX2 | PR who have developed a respiratory infection or have not gotten better |
| Mobility | 23 | MOB01 | Percent of residents who have declined in their ability to locomote |
|  | 24 | MOB1A | Percent of residents who have improved in their ability to locomote |
| Mood | 25 | MOD4A | Percent of residents who decline in mood from symptoms of depression (based on MDS Depression Rating Scale) |
| Nutrition/Weight | 26 | NUT01 | Percent of residents with a feeding tube |
|  | 27 | WGT01 | PR who have unexplained weight loss |
| Pain | 28 | PAI0X | Percent of residents with pain |
|  | 28 | PAN01 | Percent of residents with worsening pain |
| Pressure ulcers | 30 | PRU05 | Percent of residents who have a Pressure Ulcer Stage 2 to 4 |
|  | 31 | PRU09 | PR with Newly Occurring Pressure Ulcer Stage 2 to 4 |
|  | 32 | PRU04 | PR with worsening pressure sores |
| Restraints | 33 | RES01 | Percent of residents in physical restraints |
| Medication | 34 | DRG01 | PR on Antipsychotics without a diagnosis of psychosis (high & low risk) |

**Quality Indicators Currently Reported In CCRS**

| **Quality Domain** | **Quality Indicator** |
| --- | --- |
| ADL | Incidence of decline in late loss ADLs* |
| Behaviour | Prevalence of behavioural symptoms affecting others |
| Continence | Prevalence of bladder or bowel incontinence |
|  | Prevalence of occasional or frequent bladder or bowel incontinence without a toileting plan |
|  | Prevalence of indwelling catheters* |
|  | Prevalence of fecal impaction |
|  | Prevalence of urinary tract infections* |
| Cognitive function | Incidence of cognitive impairment* |
| Falls/Accidents | Prevalence of falls* |
|  | Incidence of new fractures |
| Mobility/Activity | Prevalence of little or no activity |
|  | Incidence of decline in ROM |
|  | Prevalence of bedfast residents |
| Mood | Prevalence of symptoms of depression |
|  | Prevalence of depression without anti-depressant therapy |
| Nutrition/Weight | Prevalence of weight loss* |
|  | Prevalence of tube feeding* |
|  | Prevalence of dehydration |
| Pressure ulcers | Prevalence of Stage 1-4 pressure ulcers |
| Restraints | Prevalence of daily physical restraints* |
| Medication | Prevalence of anti-psychotic drug use in the absence of psychotic and related conditions* |
|  | Prevalence of anti-anxiety/hypnotic drug use |
|  | Prevalence of hypnotic drug use more than two days in past week |
|  | Prevalence of use of 9 or more different medications |

*****Indicators included in the new proposed set

**Proposed new CCRS Quality Indicators – selected from interRAI 3^rd^ generation QIs**

| **Domain** | **#** | **Code** | **Quality Indicator** | **Numerator** | **Denominator[[1]](#footnote-2)** | **Risk Adjustment** | |
| --- | --- | --- | --- | --- | --- | --- | --- |
|  |  |  |  |  |  | **Individual Covariates** | **Facility-Level Stratification** |
| ADL | 1 | ADL01 | Percent of residents who had an unexpected loss of function in some basic daily activities | Residents with worsening (increasing item score) in Late-Loss ADL (LLADL) self-performance at target relative to prior assessment | Residents whose LLADL score can decline (do not have max score on prior assessment), excluding comatose and end-of-life residents | Age less than 65 | ADL-Long |
|  | 2 | ADL05 | Percent of residents who improve status on mid-loss ADL functioning (transfer, locomotion) or remain completely independent in mid-loss ADLs | Residents with a MLADL change score that is negative or 0 at Prior and Target assessments | Residents with valid assessments, excluding comatose and end-of-life residents | Age less than 65  CPS | ADL-Long |
|  | 3 | ADL06 | Percent of residents who improve status on early-loss ADL functioning (dressing and personal hygiene) or remain completely independent in early-loss ADLs (ELADL). | Residents with an ELADL change score that is negative or 0 at Prior and Target assessments | Residents with valid assessments, excluding comatose and end-of-life residents | RUG Late Loss ADL Scale  Age less than 65 | CPS |
|  | 4 | ADL1A | Percent of residents who had an improvement of function in some basic daily activities | Residents with improvement (decreasing item score) in Late-Loss ADL self-performance at target relative to prior assessment | Residents whose LLADL score can improve (do not have max score on prior assessment), excluding comatose and end-of-life residents | PSI: Subset 1 Diagnoses  CPS  RUG Behavior  RUG Cognitive Impairment  Age less than 65 | CMI |
|  | 5 | ADL5A | Percent of residents who declined status on mid-loss ADL functioning (transfer, locomotion) or remain completely dependent in mid-loss ADLs | Residents with a MLADL change score that is negative or 0 at Prior and Target assessments | Residents with valid assessments, excluding comatose and end-of-life residents | Not totally dependent in transferring  Locomotion problem  PSI: Subset 2 Non-Diagnoses  Age less than 65 | CMI |
|  | 6 | ADL6A | Percent of residents who declined status on early-loss ADL functioning (dressing and personal hygiene) or remain completely dependent in early-loss ADLs (ELADL). | Residents with a ELADL change score that is negative or 0 at Prior and Target assessments | Residents with valid assessments, excluding comatose and end-of-life residents | Not totally dependent in transferring  Locomotion problem  PSI: Subset 2 Non-Diagnoses  CPS  Age less than 65 | CMI |
|  | 7 | ADL7D | Proportion of ADLs changes that are decline | Residents with a valid ADL-Long Form at target assessment greater than at prior assessment | Residents with valid assessments, excluding comatose and end-of-life residents | Not totally dependent in transferring  Locomotion problem  PSI: Subset 2 Non-Diagnoses  Age less than 65 | CMI |
| Behaviour | 8 | BEHD4 | Percent of residents who have declining behavioral symptoms | Residents with more behavioral symptoms present at target assessment relative to prior assessment | Residents with valid assessments, excluding comatose residents | CPS  Motor Agitation  Age less than 65 | CPS |
| Continence | 9 | CAT02 | Percent of residents with indwelling catheters | Residents with an Indwelling catheter on target assessment | Residents with valid assessments, excluding end-of-life residents | Pressure Sore (stage 3 or 4)  ALS\MS Diagnosis  Age less than 65 | CMI |
|  | 10 | CNT02 | Percent of residents with worsening bowel continence | Residents with a value for bowel incontinence greater at target assessment relative to prior assessment | Residents with valid assessments, excluding those with maximum bowel incontinence score on previous assessment, ostomy present, comatose and end-of life residents | RUG Nursing CMI  PSI: Subset 1 Diagnoses  PSI: Subset 2 Non-Diagnoses  Age less than 65 | ADL-Long |
|  | 11 | CNT03 | Percent of residents with worsening bladder continence | Residents with a value for bladder incontinence greater at target assessment relative to prior assessment | Residents with valid assessments, excluding those with maximum bladder incontinence score on previous assessment, comatose and end-of life residents | PSI: Subset 1 Diagnoses  PSI: Subset 2 Non-Diagnoses  CPS  RUG Nursing CMI  Age less than 65 | ADL-Long |
|  | 12 | CNT04 | Percent of residents with a urinary tract infection | Residents with urinary tract infection on target assessment | Residents with valid assessments, excluding end-of-life residents | Age less than 65 | CMI |
|  | 13 | CNT2A | Percent of residents with improving bowel continence | Residents with a value for bowel incontinence less at target assessment relative to prior assessment | Residents with valid assessments, excluding those with minimum bowel incontinence score on previous assessment, ostomy present, comatose and end-of life residents | Age less than 65  PSI: Subset 1 Diagnoses | CPS |
|  | 14 | CNT3A | Percent of residents with improving bladder continence | Residents with a value for bladder incontinence less at target assessment relative to prior assessment | Residents with valid assessments, excluding those with minimum bladder incontinence score on previous assessment, comatose and end-of life residents | Age less than 65  PSI: Subset 1 Diagnoses | CPS |
| Cognitive function | 15 | COG01 | Percent of residents whose cognitive ability has worsened | Residents with score on CPS that is higher on target relative to prior assessment | Residents with valid assessments, excluding those with maximum score on Cognitive Performance Scale on previous assessment, comatose and end-of life residents | Age less than 65 | CMI |
|  | 16 | COG1A | Percent of residents whose cognitive ability has improved | Residents with score on CPS (CPS, Morris et al. 1994) that is lower on target relative to prior assessment | Residents with valid assessments, excluding those with minimum score on Cognitive Performance Scale on previous assessment, comatose and end-of life residents | Age less than 65  Full PSI  PSI: Subset 1 Diagnoses | CPS |
| Communication | 17 | COM01 | Percent of residents whose ability to communicate has worsened | Residents with a Communication Scale score (sum of 'ability to understand others' and 'making self understood‘) that is greater at the target assessment relative to the prior assessment | Residents with valid assessments, excluding those with maximum score on Communication Scale on previous assessment, comatose and end-of life residents | Short term memory problem  Long term memory problem  Age less than 65 | CPS |
|  | 18 | COM1A | Percent of residents whose ability to communicate has improved | Residents with a Communication Scale score (sum of 'ability to understand others' (C6) and 'making self understood' (C4)) that is lower at the target assessment relative to the prior assessment | Residents with valid assessments, excluding those with minimum score on Communication Scale on previous assessment, comatose and end-of life residents | Age less than 65  PSI: Subset 1 Diagnoses | CPS |
| Delirium | 19 | DELOX | Percent of residents with symptoms of delirium | Residents satisfying any of the following 3 conditions: 1) Any delirium symptom that departs from usual functioning on target assessment; 2) Any delirium symptom that departs from usual functioning on the prior assessment AND is present on the target assessment; 3) Any delirium symptom that was not present on the prior assessment AND is present on the target assessment AND the CPS (CPS) score indicates that cognitive impairment is not severe (CPS = 0, 1, 2, or 3) on the target assessment | Residents with valid assessments, excluding comatose and end-of-life residents | Age less than 65 | DRS |
| Falls | 20 | FAL02 | Percent of residents who have fallen in the last 30-days | All residents who had a fall in the last 30 days recorded on the target assessment | Residents with valid assessments | Not totally dependent in transferring  Locomotion Problem  PSI: Subset 2 Non-Diagnoses  Any Wandering  Unsteady gait/cognitive impairment  Age less than 65 | CMI |
| Infection | 21 | INF0X | Percent of residents with infections | Residents with any of the following infections or health conditions noted on the target assessment.  1. Pneumonia  2. Respiratory infection  3. Septicemia  4. Urinary tract infection  5. Viral hepatitis  6. Wound infection  7. Fever  8. Recurrent lung aspiration | Residents with valid assessments, excluding end-of-life residents | Age less than 65 | CMI |
|  | 22 | RSPX2 | PR who have developed a respiratory infection or have not gotten better | Residents with none of the selected respiratory conditions at prior assessment AND with at least one of the respiratory conditions at the Target assessment OR Residents with at least one of the respiratory conditions at the Prior assessment AND with the same or a higher count of selected respiratory conditions on the Target assessment than on the Prior assessment. The respiratory conditions included in the count (range 0 to 4) are:  1.Pneumonia  2.Inability to lie flat due to shortness of breath  3.Shortness of breath  4.Recurrent aspirations | Residents with valid assessments | RUG Clinically Complex  Age less than 65  RUG Nursing CMI | PAIN |
| Mobility | 23 | MOB01 | Percent of residents who have declined in their ability to locomote | Total number of residents whose value locomotion self-performance is greater at target relative to prior assessment | Residents with valid assessments, excluding those with a total dependence score for locomotion on unit in previous assessment, comatose and end-of-life residents | PSI: Subset 1 Diagnoses  More dependence in toileting  Requires much assistance for eating  Age less than 65 | CMI |
|  | 24 | MOB1A | Percent of residents who have improved in their ability to locomote | Total number of residents whose value locomotion self-performance is less at target relative to prior assessment | Residents with valid assessments, excluding those with independent score for locomotion on unit in previous assessment, comatose and end-of-life residents | Age less than 65  PSI: Subset 2 Non-Diagnoses  CPS  Requires much assistance for eating | CPS |
| Mood | 25 | MOD4A | Percent of residents who decline in mood from symptoms of depression (based on MDS Depression Rating Scale) | Residents who have a DRS scale change score that is positive between the Prior and Target assessments | Residents with valid assessments, excluding those with maximum Depression Rating Scale value on previous assessment | Age less than 65 | CMI |
| Nutrition/Weight | 26 | NUT01 | Percent of residents with a feeding tube | All residents with a feeding tube at target assessment | Residents with valid assessments, excluding comatose and end-of-life residents | RUG Clinically Complex  Swallowing problem  RUG Nursing CMI  Age less than 65 | ADL-Long |
|  | 27 | WGT01 | PR who have unexplained weight loss | Residents with weight loss on target assessment | Residents with valid assessments, excluding end-of-life residents and those on a planned weight-loss program | Age less than 65 | CMI |
| Pain | 28 | PAI0X | Percent of residents with pain | Residents with moderate pain at least daily or horrible/excruciating pain at any frequency on the target assessment. | Residents with valid assessments | CPS  Long term memory problem  Age less than 65 | DRS |
|  | 28 | PAN01 | Percent of residents with worsening pain | Residents with greater pain at target assessment relative to prior assessment, defined by greater score on the Pain Scale | Residents with valid assessments, excluding those with maximum Pain Scale score on previous assessment | Age less than 65 | CMI |
| Pressure ulcers | 30 | PRU05 | Percent of residents who have a Pressure Ulcer Stage 2 to 4 | All residents who had a stage 2 to 4 pressure ulcer on target assessment | Residents with valid assessments | RUG Cognitive Impairment  PSI: Subset 1 Diagnoses  More dependence in toileting  Age less than 65 | CMI |
|  | 31 | PRU09 | PR with Newly Occurring Pressure Ulcer Stage 2 to 4 | All residents who had a stage 2 to 4 pressure ulcer on target assessment and no stage 2 to 4 on prior assessment | Residents with valid assessments, excluding those with stage 2 or greater ulcer on previous assessment | Age less than 65  PSI: Subset 1 Diagnoses  More dependence in toileting  RUG Cognitive Impairment | CMI |
|  | 32 | PRU04 | PR with worsening pressure sores | Total number of residents evidencing more severe pressure ulcers on the target assessment versus the prior assessment | Residents with valid assessments | RUG Late Loss ADL  Age less than 65 | CMI |
| Restraints | 33 | RES01 | Percent of residents in physical restraints | Residents who were physically restrained daily on target assessment | Residents with valid assessments | None | ADL-Long |
| Medication | 34 | DRG01 | PR on Antipsychotics without a diagnosis of psychosis (high & low risk) | Residents receiving antipsychotics on target assessment | Residents with valid assessments and certain ICD9 psyciatric disorders, excluding those with schizophrenia, Tourette Syndrome and Huntington’s Syndrome, as well as those with hallucinations present only on target assessment. End of life residents are also excluded. | Motor Agitation  Moderate/impaired decision making problem  Long term memory problem  CPS  Combination Alzheimer’s Disease/Other Dementia  Age less than 65 | CMI |

1. In general, residents are only included in the calculation of the quality indicators if they have been in the facility long enough to have assessments in two consecutive quarters. The majority of indicators measure change in the residents’ status, which compare one quarter’s assessment (the target) with the assessment from the previous quarter (the prior assessment). Residents are only included if they have both a valid target and prior assessment. Other (prevalence) measures require only a valid target assessment. Assessments are usually excluded in they contain any missing data for the data elements used in the indicator calculations. Other indicator-specific inclusions/exclusions are listed in the table. [↑](#footnote-ref-2)
