# Supplementary material for: Understanding feedback report uptake: process evaluation findings from a 13-month feedback intervention in long-term care settings
Source: Implement Sci. 2015 Feb 12;10:20. doi: 10.1186/s13012-015-0208-2 (PMC4331147; doi:10.1186/s13012-015-0208-2)
Supplement: Additional file 4: — Observational checklist. [file 13012_2015_208_MOESM4_ESM.docx]

|  | **Month 01** | | | | **Month 02** | | | |
| --- | --- | --- | --- | --- | --- | --- | --- | --- |
|  | 20-Jan-09 | 20-Jan-09 | 22-Jan-09 | 22-Jan-09 | 17-Feb-09 | 17-Feb-09 | 19-Feb-09 | 19-Feb-09 |
|  | 10:30am-12 | 630pm-8pm | 1030am-12 | 630pm-8pm | 10:30am-12 | 630pm-8pm | 1030am-12 | 630pm-8pm |
| Staff Member Reading Report (and asking questions) |  |  |  |  |  |  |  |  |
| Staff Member Reading Report (no questions) |  |  |  |  |  |  |  |  |
| Staff member putting report in pocket/somewhere else without reading |  |  |  |  |  |  |  |  |
| Staff member throwing report away without reading |  |  |  |  |  |  |  |  |
| Staff member throwing report away after reading |  |  |  |  |  |  |  |  |
| One staff member discussing report with another staff member |  |  |  |  |  |  |  |  |
| Staff member reads and offers to put it in a team book/common area for others to see |  |  |  |  |  |  |  |  |
| Report not given directly to an individual (handout person left in a common area, under a door, etc.) |  |  |  |  |  |  |  |  |
| Other (*please specify) |  |  |  |  |  |  |  |  |
| No Observation Recorded |  |  |  |  |  |  |  |  |
| **Total** |  |  |  |  |  |  |  |  |
| *Report not given because individual already seen it (copy in team room or from another staff member)* |  |  |  |  |  |  |  |  |
| *Other: |  |  |  |  |  |  |  |  |
| Notes |  |  |  |  |  |  |  |  |
