# Supplementary material for: Understanding feedback report uptake: process evaluation findings from a 13-month feedback intervention in long-term care settings
Source: Implement Sci. 2015 Feb 12;10:20. doi: 10.1186/s13012-015-0208-2 (PMC4331147; doi:10.1186/s13012-015-0208-2)
Supplement: Additional file 5: — Additional results and findings. [file 13012_2015_208_MOESM5_ESM.docx]

**Additional results and findings**

Behaviors observed during feedback report distribution (Table 5-1)

In Table 5-1, we present counts and percentages of staff behaviors observed when reports were hand-distributed, by facility over the entire time period. The total number of reports distributed ranged from 103 in Feedback Month 12 to 229 in Feedback Month 3, although the method of delivery varied widely from 12% to 65% delivered directly to individuals and 15% to 87% left for later distribution. The proportion of reports left for later distribution increased over the period of the intervention.

Provider response to feedback reports across facilities (Figure 5-1)

In Figure 5-1, we show the proportions of staff responses to the feedback reports, reported through the post-feedback surveys, aggregated across facilities over the full 8 Survey Cycles. Notably, these trends are not monotonically increasing or decreasing for any of the uptake elements, but all varied both up and down across the survey cycles. The proportion of respondents who said they read the report was initially very high, but dropped off after Cycle 2, due to a change in the way we asked the question about how much of the feedback report they had read. It stayed between 70 and 80% after that point. Over the full period, the proportion of respondents who said that they understood the report followed a similar pattern, although this proportion dropped below 70% in Cycle 6.

The proportion of respondents who said that they found the report useful overall started relatively low (53%) and climbed through most of the intervention period to 75% in Cycle 9, with slight dips in Cycles 5 and 6. The proportion of respondents who said they discussed the report with another staff member fluctuated considerably during the entire period, with the highest point in Cycle 2 (55%), the lowest point in Cycle 5 (32%), and ending at 41% in Cycle 9. The proportion of providers who found the reports useful to make changes in the way they take care of residents stayed between 65 and 75% throughout the study.

Over time, the proportion of respondents who said they had read and understood the report remained similar to each other. The proportion of providers who found the reports generally useful increased over the intervention period, from just over 50% to about 75% from Cycle 1 to Cycle 9, while the proportion who said they discussed the reports with another staff member started relatively high (40-50% in Cycles 1 and 2), but then went up and down over the remainder of the intervention period. The proportion in Cycle 9 was similar to that in Cycle 1.

Response to survey by provider type and survey cycle (Figures 5-2 through 5-5)

*Receiving the report (Figure 5-2)*

The proportion of respondents stating that they received the report was consistently high throughout the intervention period. Note that this question was whether they had received the report during report distribution; if not, their response was recorded as “no,” but they were handed a report to look at in order to complete the remainder of the survey. The high rate of positive responses to this question indicates that providers were receiving feedback reports.

*Reading the report (Figure 5-3)*

In Cycles 1 and 2 the questions about reading and understanding were asked differently than in Cycle 3 forward, and show higher proportions across all provider types. HCAs consistently show lower proportions even in Cycles 1 and 2, but even among HCAs, the proportion reporting that they have read the entire report varies from slightly above 40% (Cycle 4) to a high of 60% in Cycle 5. Among RN/LPNs and AHPs, the proportion reporting they have read the entire report varies across the period. As with HCAs, the proportion reporting that they have read the entire report is always above 40%, with the highest proportions at 71% for AHPs in Cycle 3, and at 66% for RN/LPNs in Cycle 4.

*Understanding the report (Figure 5-4)*

As with the question about reading the report, we changed the question to obtain more nuanced information about how much of the report a respondent felt they had understood. This change had a particular effect on HCA responses. They generally reported that they understood some of the report, but relatively low proportions reported understanding all of it. As with reading the reports, there is no consistent pattern across the time period for any provider type. Generally, the proportions reporting that they understood the whole report are lower than those reporting they had read the whole report.

*Finding the report generally useful (Figure 5-5)*

In this, we report on the proportion of respondents who said that they found the report at least “somewhat” useful generally. In many cycles, at least one group of respondents all reported finding the report somewhat useful—AHPs in Cycles 1, 3, and 4; HCAs in Cycles 3, 4, and 9. In none of the cycles did 100% of the RN/LPN group say that they found the report somewhat useful, although the 99% figure in Cycle 9 is very close. The proportion finding the report at least somewhat useful did not go below 80% for any provider type in any cycle.

We provide additional graphs by provider group for the uptake scale described in the main text (Figure 5-6a-c).

*Discussing the report with another staff member (Table 5-2, Figures 5-7 through 5-9)*

We provide a number of tables and graphs in this area because of our interest in understanding how much interaction there was among staff members in discussing the feedback reports, as an indicator of social interaction and influence. Even though we provided the reports individually to providers, our expectation was that they would discuss them with other staff members.

In Table 5-2 we provide the number of respondents by provider type who said that they discussed the feedback report with another staff member by cycle. Discussing the reports appeared more common during the beginning of the intervention, less so during the middle of the intervention, with an increase again towards the end. AHPs appeared less likely to discuss the reports with another staff member than others.

In Figure 5-7, we provide information about what respondents said they talked about if they discussed the reports, for Cycles 1 and 9. We changed the question somewhat after Cycle 2, and no longer asked if they discussed ideas for resident care with another staff member, retaining two specific responses (to find out what others thought, and to get advice about resident care) and one “other” category. In both Cycle 1 and Cycle 9, more than half of respondents said that they discussed the reports to find out what others thought about them; a smaller proportion said that their discussion was to get advice; and in Cycle 1 only, over half of respondents said that they discussed the reports to get ideas about resident care from other staff, whereas in Cycle 9, none of the respondents gave this as the reason for discussing the reports. Comparing across units or facilities is mentioned, as is understanding the reports, or checking with others to see if the respondent understood the report. RNs, LPNs and unit Care Managers are the group who most often reported “other” reasons for discussion, not surprising given their role as supervisors of HCAs and other staff. HCAs rarely gave any other reason for discussion, while AHPs often did.

Figures 5-8 and 5-9 provide detail by provider type across the cycles about the proportion of respondents who reported discussing the feedback reports to get others’ thoughts, and to get advice about resident care.

In Table 5-4, we present information collected only in Cycle 9 looking back over the past year while the feedback intervention was going on, describing how many and what proportion of survey respondents said that they had discussed the feedback reports in a staff meeting, and how often, by provider type. In this table, we provide information on Care Managers, not grouped in with RNs and LPNs. All three Care Managers responding to the survey (out of a possible 9) said that they had discussed the feedback report during a staff meeting in the last year, with two thirds saying they had discussed it between 1 and 3 times, and one third saying they had discussed it between 3 and 6 times. AHPs were the next most likely to say that they had discussed the feedback reports during staff meetings (56%), with 80% saying they had discussed it between 1 and 3 times, and 10% each reporting between 3 and 6 or more than 6 times. Interestingly, RN/LPNs were least likely to say that they had discussed the feedback reports in a staff meeting (46%). Two-thirds reported discussing them 1-3 times, and 26% said they had discussed them between 3 and 6 times. Almost half of HCA respondents said they had discussed the reports in a staff meeting, with almost 90% reporting they did so 1 to 3 times in the last year.

Finding the report useful to change resident care (Figure 5-10)

This specific question about the usefulness of the reports for the purpose of changing the way providers care for residents varied in the responses to it by cycle and by provider type. HCAs generally, across all cycles, reported finding the reports useful in changing how they provided care to residents, although the proportion peaked in Cycles 3-5 and dropped to about 55% by Cycle 9. RNs and LPNs also reported finding the report useful for changing resident care most often in the middle of the period, and dropped off to 57% in the last cycle. AHPs started off reporting in high proportions (67-82% for Cycles 1-3) that they found the reports useful, but dropped off considerably in Cycles 4-6 (52-42%), with some increase in Cycles 7/8 and 9.

**Additional File 5: Figure Legend**

Figure 5-1: Responses to items measuring uptake of feedback reports over the 9 Survey Cycles for all providers

Figure 5-2: Respondents who report receiving report by provider type and cycle

Figure 5-3: Respondents who report reading the report by provider type and cycle

Figure 5-4: Respondents who report understanding the report by provider type and cycle

Figure 5-5: Respondents who report finding the feedback report at least “somewhat” useful by provider type and cycle

Figure 5-6a-c: Uptake scale by provider group Cycles 3-9

Figure 5-7: What respondents talked about in discussing feedback reports with other staff, at beginning (Cycle 1) and end (Cycle 9) of the intervention

Figure 5-8: Proportion of respondents who discussed to get others’ thoughts about the feedback report by provider type and cycle

Figure 5-9: Proportion of respondents who asked for advice on resident care in discussing feedback reports by provider type and cycle

Figure 5-10: Respondents who report finding the report useful in changing resident care by provider type and cycle

**Additional File 5: Tables**

| Actions observed | Overall | Facility 1 | | Facility 2 | | Facility 3 | | Facility 4 | |
| --- | --- | --- | --- | --- | --- | --- | --- | --- | --- |
|  | (N=2365) | (n=759) | | (n=662) | | (n=625) | | (n=319) | |
|  | No. | No. | % | No. | % | No. | % | No. | % |
| Staff member reading report and asking questions | 104 | 22 | 2.9 | 27 | 4.1 | 29 | 4.6 | 26 | 8.2 |
| Staff member reading report but no questions | 342 | 118 | 15.6 | 94 | 14.2 | 73 | 11.7 | 57 | 17.9 |
| Staff member putting report in pocket or somewhere else without reading | 268 | 56 | 7.4 | 74 | 11.2 | 66 | 10.6 | 72 | 22.6 |
| Staff member throwing report away without reading | 1 | − | − | − | − | 1 | 0.2 | − | − |
| Staff member throwing report away after reading | − | − | − | − | − | − | − | − | − |
| One staff member discussing report with another staff member | 12 | 5 | 0.7 | − | − | 5 | 0.8 | 2 | 0.6 |
| Staff member offers to put it in team book or a common area (e.g. bulletin board) for other staff members to see | 59 | 10 | 1.3 | 32 | 4.8 | 13 | 2.1 | 4 | 1.3 |
| Report not given directly to an individual (e.g. left in a common area, under a door or in a mailbox) | 1518 | 540 | 71.2 | 414 | 62.5 | 411 | 65.8 | 153 | 48.0 |
| Others (e.g. refused to receive the report) | 61 | 8 | 1.1 | 21 | 3.2 | 27 | 4.3 | 5 | 1.6 |

Table 5-1: Behaviors observed at feedback distribution by facility throughout the intervention period

| Provider type | Cycle 1 | Cycle 2 | Cycle 3 | Cycle 4 | Cycle 5 | Cycle 6 | Cycle 7/8 | Cycle 9 |
| --- | --- | --- | --- | --- | --- | --- | --- | --- |
| HCA | 26 | 20 | 21 | 27 | 16 | 24 | 38 | 44 |
| RN/LPN | 17 | 21 | 10 | 12 | 13 | 11 | 24 | 26 |
| Allied Health professionals | 11 | 8 | 8 | 8 | 3 | 3 | 9 | 5 |

Table 5-2: Number of providers by type who discussed reports with other staff members Cycles 1 to 9

| **Other reason for discussing with another staff member** | **Provider type** |
| --- | --- |
| **Cycle 1 (N = 10)** | |
| Compare to current RAI reports | RN/LPN/CM |
| Compare to statistics from other facilities | RN/LPN/CM |
| Discuss unit participation | RN/LPN/CM |
| Following up on information in report | RN/LPN/CM |
| To discuss if it really will make changes | HCA |
| Discuss what the graphs mean | AHP |
| Discuss how to read the report | AHP |
| On agenda for MDS meeting | AHP |
| To understand more about long term care | AHP |
| To understand the report and what's happening on the unit | AHP |
| **Cycle 2 (N = 9)** | |
| Shocked at some of the site demographics | RN/LPN/CM |
| Questions about determining staffing | RN/LPN/CM |
| Comparing to other facilities | RN/LPN/CM |
| Discuss how the report is useful for resident care | RN/LPN/CM |
| Exchanging ideas | RN/LPN/CM |
| Discussed at MDS meeting | AHP |
| Brainstorming how to interpret graphs | AHP |
| Consider making recommendations | AHP |
| Classifying units | AHP |
| **Cycle 3 (N = 2)** | |
| Discussed methods to improve resident care | RN/LPN/CM |
| Compared results from previous reports | RN/LPN/CM |
| Sharing information about the reports | AHP |
| **Cycle 4 (N = 5)** | |
| Understand what the other staff member thinks about the report | RN/LPN/CM |
| Get ideas for addressing issues | RN/LPN/CM |
| Check to make sure I understood the report | AHP |
| Wanted to share because of the issues it raised | AHP |
| **Cycle 5 (N = 4)** | |
| Information about the study and why it's being done | RN/LPN/CM |
| To see if other staff understand the reports | RN/LPN/CM |
| Get help to understand the report | HCA |
| To debate the validity of the data | AHP |
| To discuss interpretations of the data | AHP |
| **Cycle 6 (N = 4)** | |
| To understand the report | RN/LPN/CM |
| To discuss MDS with registered nurses and see about appropriate action | RN/LPN/CM |
| Wanted to read the whole report before discussing | HCA |
| **Cycle 7/8 (N = 13)** | |
| Discussion about pain in residents | RN/LPN/CM |
| Curious to see if others understood it | RN/LPN/CM |
| Interesting to see where we are | RN/LPN/CM |
| Discuss type of residents on unit | RN/LPN/CM |
| Discuss best practices | RN/LPN/CM |
| To make sure the other person understands the results | RN/LPN/CM |
| Why the unit is so much higher on some measures | RN/LPN/CM |
| To see if others want to discuss it | RN/LPN/CM |
| Interesting to see how we compare | RN/LPN/CM |
| For my safety and the safety of residents | HCA |
| No time to discuss due to increased workload | HCA |
| To understand the reports better | HCA |
| Clarifying the report | AHP |
| Reasons for discrepancies | AHP |
| **Cycle 9 (N = 7)** | |
| Just discussing | RN/LPN/CM |
| Talked about comparing | RN/LPN/CM |
| To see how we're doing | RN/LPN/CM |
| We talked a little bit | HCA |
| To make suggestions so the report can be more informative | HCA |
| To discuss the current details | AHP |

Table 5-3: Other reasons given by respondents who discussed reports with another staff member, Cycles 1 to 9

| **Provider type** | **Discussed at least once** | **Discussed at least once** | **Number of times discussed in last year** | | |
| --- | --- | --- | --- | --- | --- |
|  | **Number yes** | **Percent of respondents** | **1 to 3** | **3 to 6** | **More than 6** |
| **Care Manager** | 3 | 100% | 67% | 33% | 0% |
| **RN/LPN** | 27 | 46% | 67% | 26% | 7% |
| **HCA** | 52 | 48% | 88% | 8% | 4% |
| **AHP** | 10 | 56% | 80% | 10% | 10% |
| **Total** | 92 | 48% | 81% | 14% | 5% |

Table 5-4: Responses to “Did you discuss feedback reports during a staff meeting in the last year?” and number of times discussed in staff meeting by provider type (Cycle 9 only)

**Figures**

Figure 5-1: Responses to items measuring uptake of feedback reports over the 8 Survey Cycles

Note: In this figure, we show the proportion of respondents who said that they read and/or understood at least half of the feedback report from Cycle 3 forward when the questions were changed

Figure 5-2: Respondents who report receiving report by provider type and cycle

Figure 5-3: Respondents who report reading the report by provider type and cycle

*In Cycles 1 and 2, respondents answered “Did you read the report?” with a yes/no response. From Cycle 3 on, they were asked to say whether they read less than half, half, more than half, or the entire report. In this graph, we report on the proportion who said they read the entire report.

Figure 5-4: Respondents who report understanding report by provider type and cycle

*In Cycles 1 and 2, respondents answered “Did you understand the report?” with a yes/no response. From Cycle 3 on, they were asked whether they understood less than half, half, more than half, or the entire report. In this graph, we report on the proportion who said they understood the entire report.

Figure 5-5: Respondents who report finding the feedback report at least “somewhat” useful by provider type and cycle

Figure 5-6a: Uptake scale for Health Care Aides

*From Cycle 3 on, we asked if respondents had read less than half, half, more than half or all of the report. The percentage reported is those that stated they read the entire report. The same is true of the question about understanding the report.

Figure 5-6b: Uptake scale for RNs and LPNs

*From Cycle 3 on, we asked if respondents had read less than half, half, more than half or all of the report. The percentage reported is those that stated they read the entire report. The same is true of the question about understanding the report.

Figure 5-6c: Uptake scale for Allied Health Professionals

*From Cycle 3 on, we asked if respondents had read less than half, half, more than half or all of the report. The percentage reported is those that stated they read the entire report. The same is true of the question about understanding the report.

Figure 5-7: What respondents talked about in discussing feedback reports with other staff, at beginning (Cycle 1) and end (Cycle 9) of the intervention

Figure 5-8: Proportion of respondents who discussed to get others’ thoughts about the feedback report by provider type and cycle

Figure 5-9: Proportion of respondents who asked for advice on resident care in discussing feedback reports by provider type and cycle

Figure 5-10: Respondents who report finding the report useful in changing resident care by provider type and cycle
